# Pro-Donation Behaviours of Nursing Students from the Four Countries of the UK

**DOI:** 10.1371/journal.pone.0091405

**Published:** 2014-03-10

**Authors:** Donal McGlade, Carol McClenahan, Barbara Pierscionek

**Affiliations:** 1 Faculty of Science, Engineering and Computing, Kingston University London, Kingston upon Thames, England, United Kingdom; 2 School of Psychology, University of Ulster, Coleraine, Northern Ireland, United Kingdom; University of Colorado, United States of America

## Abstract

**Background:**

The effectiveness of the organ donation system depends on the health professionals involved in procurement and in dealing with donors and their families. Concerns about lack of knowledge and experience of organ donation have been expressed among such professionals but there is a paucity of literature to indicate the basis of such concerns and where knowledge may be lacking. Given that regional variations in organ donation rates exist in the UK, this study investigates knowledge about and attitudes towards organ donation among student nurses in different countries of the UK and examines regional variations.

**Methods:**

A questionnaire was distributed to 667 student nurses (female:male = 582∶85) aged 18 to 50 years (mean [SD] 25.4 [7.1] years) recruited from a total of five Universities (Scotland, Wales, Northern Ireland, England) during the period of January to September 2011.

**Results:**

Registration behaviour among participants was shown to vary depending upon many different factors that include birthplace, residency, fear of death and concerns of medical distrust.

**Conclusions:**

Regional variations in organ donation behaviour in the UK were found in the cohorts of student nurses who participated in this study. These variations include willingness to register and to donate specific body parts and not others. The relationship between attitude and behaviour and how this may influence the decision making process of organ donation, as well as the underlying factors that result in regional variations, require further investigation.

## Introduction

Organ transplantation is an integral component of modern health care practice and is an important option in the treatment of patients diagnosed with end-stage organ failure [1]. In the UK, organ donation and procurement is managed predominantly by nurses [2]–[3] and nurses are generally relied upon to identify potential donors [4], [5]. Indeed, since 2009, in the UK the pool of donor coordinators has increased and specialist nurses for organ donation (SNOD) are now integral to all intensive care units in the UK [6]. Yet, findings suggest that nurses commonly exhibit concerns about their lack of knowledge and experience in dealing adequately with all aspects of organ donation and transplantation [4], [7]–[13].

In a UK-based study of student nurses, the findings revealed that 99% of respondents supported organ donation and could recognise the beneficence associated with the act of donation [14]. However, this did not necessarily translate into a personal willingness to donate organs [14]. It was found that only 74% (n = 26) demonstrated a commitment to organ donation and had registered as an organ donor [14]. The study also highlighted a reluctance to donate specific body parts, with 14% of student nurses indicating that they would refuse to donate their corneae [14]. Similar reservations have been expressed by other UK nurses, with 25% (n = 28) averse to corneal donation [5].

Given the fundamental role that nurses play in the organ donation system in the UK, which involves raising the prospect of organ donation with potential donors and their families, explaining the process and obtaining consent, appropriate training is needed to help nurses understand and communicate the process of organ donation to potential donors and relatives. This is most pertinent as it has been shown that nurses feel unsure about how to broach a subject as delicate as organ donation during times of grief [9]–[10]. It has been suggested that reluctance to raise the question of organ donation may be related to nurses’ inability to encourage and engage patients and their relatives in the decision-making process which may stem from a lack of training and confidence in communicating the process and importance of organ donation to patients and their relatives [15]–[16]. It is plausible to suggest that encouraging nurses to develop a closer relationship with donors and their families and equipping them with necessary skills on how to sensitively broach the subject of organ donation will allow them to interact more appropriately with the family of the deceased and obtain higher consent rates for organ donation [17]–[22]. In addition to appropriate skills and knowledge, significant correlations were found between requests for organ donation and nurses’ beliefs about the benefits of donating organs and whether they were confident in their ability to request a donation [23]. Those with a personal interest in organ donation, who had registered and/or discussed their own donation intentions were more confident in making requests for organs and more successful at obtaining consent [23]–[24]. Indeed, successful procurement was attributed to a positive attitude rather than to a sound knowledge base [24].

When considering how UK-based nurses perceive and approach organ donation, it is important to recognise that the UK is made up of four distinct regions with differences in socioeconomic and demographic factors [25], proportion of different ethnic groups [26], mortality statistics [27] and in healthcare provision and education systems [28]. When the organ donation rates in the four regions were examined, significant variations in rates and in types of organs donated were found, some of which were likely to be associated with traditional values and cultural beliefs [29]. Regional variations in organ donation between kidney retrieval units across the UK have also been noted and a correlation was found between donor rate and intensive care unit bed numbers, as well as between donor rates and proportion of the population from minority ethnic groups [26].

Whilst differences in donation rates have been found in the four countries of the UK, the underlying reasons are not fully understood and need further investigation [26], [29]. One unexplored factor is the knowledge and attitude of student nurses in the four regions. A student population is most likely to provide responses that are as yet unaffected by experiences in professional practice and therefore most likely to reflect underlying beliefs of the individual. This study aimed to examine the knowledge and attitudes of student nurses from the four UK countries to see if any variations existed and if so, whether these were worthy of further investigation that could lead to an eventual improvement in donation rates across the UK.

## Methods

This study was approved by the Ethics Filter Committee of Biomedical Sciences at the University of Ulster. All consent was fully informed with participants given an information sheet that described the research, its aims and objectives, the role of participants, how the results would be disseminated and that all data would be anonymised. All consent obtained was in written form.

### Participants and Methods

The study was designed as a questionnaire-based analysis and was conducted in five Universities that provide training to nurses. The five Universities that gave permission for participant recruitment in the study were comprised of one Welsh, one Scottish and one Northern Irish University and two English Universities: one from the north and one from the south of the country. Sample size was determined by statistical power analysis using G*Power Version 3.13 and adopted a small (0.15) effect size [30], 0.01 level of significance and df = 3. Based upon this, a total sample size of approximately 680 participants was required in order to give an adequate (0.80) level of power [31].

The researcher (DM) visited each University to explain the nature of the study and to distribute the questionnaire and information sheet in a classroom setting with student cohorts gathered during the period of January to September 2011 using convenience sampling. Participants were pre-registered nursing students undertaking a full-time general degree course leading to the award of BSc. (Hons). Participation in the study was voluntary and without any form of compensation.

### The Questionnaire

The questionnaire was developed and pilot tested using a framework based on the topics investigated in previously published studies [5], [32]–[34]. The questionnaire has been extended to include other important concepts: attitude to registration and donation, reasons for unwillingness to register and to donate, benefits of donation, knowledge of brain death and legislation. It consisted largely of a range of closed questions that required the participant to respond using a dichotomous, polytomous or a four-point forced-choice Likert-type scale. The participant was also given the opportunity to write a free-text response where appropriate. Demographic information relating to gender, age, birthplace, residency, marital status and religious affiliation was obtained. In addition, factual based information was gathered using 23 items, with knowledge and attitude assessed using 8 and 13 items respectively (Table 1).

**Table 1:** Key questionnaire items.

**Factual items**
Have you registered to be an organ donor? (yes/no)
Why would you be unwilling to donate your eyes? (you do not want to donate a part of you which can be seen on another person/the eye embodies character and resembles deeper meaning/eye is characteristic and associated with identity)
Why would you be unwilling to donate your heart? (associated with feelings and emotions/another person may imitate my characteristics/other)
Have you discussed your organ donation intentions with your family? (yes/no)
**Knowledge items**
Does your religion allow organ donation? (yes/no/I do not know)
Which can be donated after death? (eyes/heart/kidneys/liver/lungs)
Are you aware of any laws that control organ donation? (yes/no/I do not know)
Would you consider a person who is declared brain dead but still has a beating heart as being dead? (yes/no/I do not know)
How likely do you think it is that a brain dead person with a beating heart might recover and live? (very likely/likely/unlikely/very unlikely)
**Attitudinal items**
Which organ are you willing to donate? (eyes/heart/kidneys/liver/lungs)
Becoming an organ donor makes me think about my own death? (strongly agree/agree/disagree/strongly disagree)
By signing a donor card, doctors might do something to me before I am really dead? (strongly agree/agree/disagree/strongly disagree)
The government should provide financial help to families who donate (strongly agree/agree/disagree/strongly disagree)

## Results

A total of 795 questionnaires were distributed amongst the five Universities, generating 667 completed questionnaires and a final response rate of 83.9% (England = 252; Northern Ireland = 174; Wales = 137; Scotland = 104). In some cases, not all questionnaire items were fully or clearly completed and were reported as missing data. Of those completing the questionnaire, 98.1% (n = 654) had no missing values, 1.8% (n = 12) had no more than one missing value and 0.2% (n = 1) had two missing values present. All questionnaires have been included in the analysis. Descriptive statistics were used to assess demographic information, with Pearson’s Chi-square test used to explore group differences and relationships among categorical variables using the Statistical Package for Social Sciences Version 19. Data have been made publically available from the Dryad digital repository.

Participants were predominantly female (87.3%, n = 582) reflecting the gender base of the profession [35]. A breakdown of gender by region indicated that Scotland had the greatest proportion of male participants (38.8%, n = 33) and Wales the lowest (14.1%, n = 12). Ages ranged from 18 to 50 years (mean [SD] 25.4 [7.1] years). The majority of participants (78.5%, n = 521) were under 30 years of age. Religious affiliation was found in 64.5% (n = 430) of cases. Those with a religious affiliation were predominantly Christian (97.4%, n = 419). A breakdown of religious beliefs by region showed that Northern Ireland had the highest rate of religious participants (97.1%, n = 169) and Wales the lowest (56.9%, n = 78). Further analysis of the non-Christian group was not possible due to a diverse number of faiths representing a small number of participants.

### Willingness to Register

Findings from the questionnaire show that almost half of the participants were registered as organ donors: 46.8% (n = 312). Of those not currently registered, 58.0% (n = 206) were willing to consider registration, compared with 28.2% (n = 100) who were undecided and 13.8% (n = 49) who would not consider it at all. The results indicate that willingness among participants to register correlated with where they were born (χ^2^ = 12.28, df = 4, p = 0.015) and where they were currently living (χ^2^ = 15.44, df = 3, p = 0.001) (as shown in Table 2). Participants born in England were 1.8 times more likely to have registered as an organ donor compared to those born in Northern Ireland (95% CI = 1.2 to 2.7). With regards to region of residence, participants living in Scotland were 2.4 times more likely to have registered as an organ donor compared to those in Northern Ireland (95% CI = 1.4 to 3.9). There was very little variation between England and Wales.

**Table 2:** Relationship between registration and participants’ birthplace and residency.

	Birthplace %		Residency %	
	*Registered*	*Not registered*	*Registered*	*Not registered*
England	52.2	47.8	50.0	50.0
Scotland	46.8	53.2	55.8	44.2
Wales	50.8	49.2	49.6	50.4
Northern Ireland	37.9	62.1	34.5	65.5
Outside UK	33.3	66.7	N/A	N/A
	χ^2^ = 12.28, df = 4, p = 0.015		χ^2^ = 15.44, df = 3, p = 0.001	

### Willingness to Donate Specific Organs

The proportion of participants willing to donate specific organs is shown in Table 3. Corneal tissue was found to be the least likely to be donated; just over half of the participants registered as organ donors (n = 179) and a fifth of those who were unregistered (n = 70) were prepared to donate corneal tissue (Table 3). For all other organs, willingness to donate was expressed by over 90% of the participants; that for the heart slightly lower than for kidney, liver or lung. The organs that participants were most willing to donate were the kidneys. Based on the odds ratio, participants were 114.8 times more likely to donate their kidneys than their corneae (95% CI = 28.2 to 471.0). The most common reasons given for unwillingness to donate corneal tissue were that eyes were considered to hold “distinctive characteristics” in 62.4% of cases (n = 83), they “resembled a deeper meaning” in 48.9% of cases (n = 65) and/or because “the eye can be physically seen on another person” in 22.6% of cases (n = 30).

**Table 3:** Relationship between registration and willingness to donate specific organs.

	*Total sample %*	
**Kidney**	*Registered*	*Not registered*
*Willing to donate*	99.4	57.2
*Unwilling to donate*	0.6	42.8
	χ^2^ = 166.34, df = 1, p<0.001	
**Liver**	*Registered*	*Not registered*
*Willing to donate*	98.7	56.9
*Unwilling to donate*	1.3	43.1
	χ^2^ = 161.34, df = 1, p<0.001	
**Lung**	*Registered*	*Not registered*
*Willing to donate*	98.7	52.7
*Unwilling to donate*	1.3	47.3
	χ^2^ = 183.94, df = 1, p<0.001	
**Heart**	*Registered*	*Not registered*
*Willing to donate*	93.3	49.9
*Unwilling to donate*	6.7	50.1
	χ^2^ = 149.48, df = 1, p<0.001	
**Cornea**	*Registered*	*Not registered*
*Willing to donate*	57.4	19.7
*Unwilling to donate*	42.6	80.3
	χ^2^ = 100.63, df = 1, p<0.001	

Participants born in Northern Ireland were significantly less inclined to donate heart and lung than their Scottish counterparts (χ^2^ = 4.96, exact p = 0.039). With regard to lung donation, there was significantly less willingness to donate from Northern Irish participants compared to those born in England (χ^2^ = 10.47, exact p = 0.006) and Wales (χ^2^ = 4.74, exact p = 0.044). There were no statistically significant differences between birthplace for the other organs examined. Correlation between residency and willingness to donate was only significant for the lung: there were fewer intentions to donate lung from those resident in Northern Ireland than from England (χ^2^ = 8.59, exact p = 0.010) and Wales (χ^2^ = 4.68, exact p = 0.046).

### Harbouring Fears and Distrust

The majority of participants (61.6%, n = 411) associated the process of registering as an organ donor with a fear of contemplating death (χ^2^ = 43.33, df = 1, p<0.001). Participants were 2.9 times more likely to register if they did not fear death (95% CI = 2.1 to 4.0). There was no association between age and religious beliefs or the likelihood of fearing death. Neither birthplace nor residency was associated with fear of death.

Registration was shown to be negatively affected if participants held higher levels of distrust (χ^2^ = 37.94, df = 1, p<0.001) or had concerns that their organs might be misused after death (χ^2^ = 61.51, df = 1, p<0.001). Although a proportion of participants (7.1%, n = 22) did exhibit a degree of distrust, they were nevertheless prepared to register as an organ donor. Participants who had trust in those working in the organ donation system were 4.4 times more likely to register than were those who harboured a degree of distrust (95% CI = 2.6 to 7.1). With regards to concerns about misuse, 27.6% (n = 86) of participants were prepared to support the organ donation system even though they expressed concerns that their organs might be misused after death. No association was found between birthplace or residency and the exhibition of higher levels of distrust or greater concerns about organ misuse after death.

### Support for Financial Incentives

The majority (76.6%, n = 511) of participants agreed that direct financial support should not be offered for the donation of organs. However, around one-fifth (23.4%, n = 156) indicated that they would be willing to consider certain types of incentives. From this group, charitable donations (84.6%, n = 132) and help towards funeral expenses (74.4%, n = 116) were the most popular options. The least popular incentive was a cash payment (65.4%, n = 102). Over half of the participants who were firmly opposed to financial support indicated that such incentives would be detrimental to registration (59.7%, n = 305).

The results demonstrated a significant relationship between support for financial incentives and birthplace and support for financial incentives and region of residency (Table 4). Participants born in Northern Ireland were 4.2 times (95% CI = 1.8 to 9.8) more likely to approve of financial incentives than those born in Scotland (χ^2^ = 5.03, df = 1, p = 0.025) and those resident in Northern Ireland were 2.5 times more likely to approve of financial support than those resident in Scotland (χ^2^ = 8.46, df = 1, p = 0.004) and England (χ^2^ = 5.48, df = 1, p = 0.019). No statistically significant differences were observed between the other regions.

**Table 4:** Relationship between financial incentives and participants’ birthplace and residency.

	Birthplace %		Residency %	
	*Incentives*	*No incentives*	*Incentives*	*No incentives*
England	20.7	79.3	21.03	79.0
Scotland	14.3	85.7	15.4	84.6
Wales	22.6	77.4	24.1	75.9
Northern Ireland	27.6	72.4	31.0	69.0
**Interaction**				
England * Scotland	χ^2^ = 1.60, df = 1, p = 0.206		χ^2^ = 1.50, df = 1, p = 0.220	
England * Wales	χ^2^ = 0.17, df = 1, p = 0.679		χ^2^ = 0.48, df = 1, p = 0.488	
England * Northern Ireland	χ^2^ = 2.49, df = 1, p = 0.115		χ^2^ = 5.48, df = 1, p = 0.019	
Scotland * Wales	χ^2^ = 2.09, df = 1, p = 0.148		χ^2^ = 2.76, df = 1, p = 0.096	
Scotland * Northern Ireland	χ^2^ = 5.03, df = 1, p = 0.025		χ^2^ = 8.46, df = 1, p = 0.004	
Wales * Northern Ireland	χ^2^ = 0.89, df = 1, p = 0.346		χ^2^ = 1.84, df = 1, p = 0.175	

## Discussion

The main findings of this study indicate that nearly half of the student cohort had registered as organ donors and that a further third would be willing to consider donation. The results from this study also indicate that registration varies depending upon the country of the UK in which the participant was born and the country of residence at the time the study was conducted. Participants born in England tended to be more favourably disposed towards registration than those born in Northern Ireland. Registration was also more likely to occur for those participants currently living in Scotland. Participants living in Northern Ireland appeared to have the least favourable attitude with regards to registration. This effect has been observed previously [29] and may reflect attitudes based on culture and tradition as well as on lack of awareness of what donation involves [31].

The findings substantiate previous results that show discrepancies in attitude between cohorts of student nurses [14], [36]–[38]. Participants also showed varying levels of support for the donation of different organs which supports previous work that demonstrated the existence of regional variations in donation according to organ type [29]. Willingness to donate heart and lungs varied with regard to birthplace and residency, with the greatest reluctance to donate expressed from those born and resident in Northern Ireland. This study also confirms the relatively high degree of reluctance to donate corneal tissue [5], [14], [39]–[40]; however, the results did not show significant regional differences. Reluctance to donate tissue from the eye has been attributed to issues surrounding reification and the eyes being commonly referred to as having a “window on the soul” [41]. Issues of reification were also found in relation to the heart and are thought to be based on a tendency to associate this organ with feelings and emotions [41].

Although there was no association between religious beliefs and the likelihood that participants would fear death, registration in this study was found to be negatively affected by a fear of contemplating death. Studies have shown that high levels of fear of or anxiety about death can result in a reluctance to participate in certain activities which include organ donation registration [42]–[44]. In addition, it has been suggested that difficulty in thinking about death can prevent performance of certain behaviours that fundamentally require an individual to consider their own mortality [45].

A proportion of participants in this study raised concerns about medical distrust and misappropriation of organs. This had implications about their own likelihood of registering as an organ donor and could ultimately affect their ability to engage with potential donors and their relatives in an effective manner [46]–[47]. Interestingly, a small sub-group of participants, who reported concerns, were nevertheless still prepared to register their consent. This may result from altruistic tendencies that are inherent in students who contemplate a career in a caring profession such as nursing [48].

The majority of participants disapproved of financial support and considered organ donation to be a voluntary and altruistic act. Offering money was the least popular incentive for encouraging organ donation. Such an incentive may be seen by many as coercive and thereby undermining the altruistic act of organ donation. This is consistent with the findings of Bénabou and Tirole [49] who found that the use of incentives ultimately spoils the reputational value of good deeds and casts doubt on the motive. Although the results indicate that registration would be negatively affected if financial support were to be introduced, participants did appear to react more positively towards those incentives that were considered to maintain the ethos of organ donation and which appeared less like a business transaction. Most participants expressed a preference for charitable donations, with some preferring help towards potential funeral costs or a reduction in life insurance premiums. Those not currently registered as organ donors were more receptive to financial support than those who were already registered. It may be that for those less willing to donate, the act of donation was seen more as a contractual act and therefore worthy of some form of reimbursement. The results also show that support for the introduction of financial incentives varied with birthplace and residency. Participants born in Northern Ireland and/or who were resident there expressed the most favourable attitude towards the possible introduction of financial incentives than those from other the countries of the UK.

Whilst willingness to register will be influenced by what the individual considers to be morally appropriate, personally acceptable and socially desirable, decisions can be influenced by the beliefs and intentions of family members or others in the community. This study utilised a convenience sample and it is recognised that the use of this strategy to recruit participants limits the ability to make generalisations about the total student nursing population from which the sample was chosen. Hence, it cannot be claimed that the findings are definitively representative of each country but that regional differences are worthy of further exploration.

Nurses have indicated that they have limited knowledge about organ donation [4], [7]–[12] as very little time is dedicated to teaching about this topic within the core curriculum [50]. Providing nurses with information about how to care for potential organ donors and more knowledge about the neurological assessment and management of brain injury, as well as medical diagnosis and legal implications of brain death, would help them to communicate more effectively with potential donors and their families. Ultimately there may be a need for health and educational authorities in each UK country to adapt teaching about organ donation and transplantation within core curricula so as to address any regional differences in knowledge and attitude. The differences seen in the attitudes of student nurses from the four countries of the UK may also exist in the respective wider communities. In such case, campaigns currently used to raise the public profile of organ donation in the UK [51] may be too general in context. More regionally relevant campaigns that address prevailing attitudes in each particular country of the UK may have greater effect. Further research to explore regional attitudes and donation behaviours and their underlying reasons is needed to inform such campaigns.
